# Effectiveness and safety of massage for chronic pain in patients with knee osteoarthritis: A protocol for systematic review and meta-analysis

**DOI:** 10.1097/MD.0000000000028533

**Published:** 2022-01-21

**Authors:** Jianghan Xu, Boyi Wu, Shengji Xie, Guanghui Wu, Heng Zhang, Yangyang Fu, Guangxin Guo

**Affiliations:** a School of Acupuncture-moxibustion and Tuina, Shanghai University of Traditional Chinese Medicine, Shanghai, China; b Yueyang Hospital of Integrated Traditional Chinese and Western Medicine, Shanghai University of Traditional Chinese Medicine, Shanghai, China; c Shanghai Municipal Hospital of Traditional Chinese Medicine, Shanghai University of Traditional Chinese Medicine, Shanghai, China.

**Keywords:** chronic pain, knee osteoarthritis, massage, meta-analysis, protocol

## Abstract

**Background:**

Chronic pain (CP) is a common and debilitating symptom in patients with knee osteoarthritis (KOA). Massage has been supported as a non-pharmacological intervention for the individual symptom relief of CP. However, relevant evidence of using massage for CP in patients with KOA has been lacking.

**Methods:**

A systematic search will be performed in the following electronic databases for randomized controlled trials to evaluate the effectiveness and safety of massage for CP of KOA: China National Knowledge Infrastructure (CNKI), Wan Fang, PubMed, EMBASE, PsycINFO, and the Cochrane Library databases from their inception to December 2021. The entire process will include study selection, data extraction, risk of bias assessment and meta-analysis.

**Results:**

This proposed study will evaluate the effectiveness and safety of massage for CP in patients with KOA. Changes in pain relief and adverse effect will be included in our outcomes.

**Conclusions:**

This systematic review will provide evidence for assessing the credibility of massage for CP in patients with KOA.

**Dissemination and ethics:**

The results of this review will be disseminated through peer-reviewed publication. This review does not require ethical approval because all of the data used in this systematic review and meta-analysis have already been published. Furthermore, all of these data will be analyzed anonymously during the review process.

## 1 Introduction

Knee osteoarthritis (KOA) is a common and disabling condition that typically manifests as attacks of pain around the joints, and it is a typical disease which can develop chronic pain (CP).^[1–3]^ The morbidity of KOA is quite high among the old. It is reported that 30% to 50% of people over the age of 60 suffer from KOA.^[4]^ Such a high incidence leads to a huge personal and socio-economic burden.^[5]^ The patient's ability to move is limited by CP,^[6]^ which further leads to the aggravation of pain symptoms.^[7]^ CP is one of the most common symptoms in patients with KOA.^[8]^ 25% of KOA patients suffer from severe joint pain.^[9]^ CP is defined as pain that lasts more than 3 to 6 months.^[10–12]^ Patients with CP are more likely to suffer from depression, anxiety, and insomnia.^[13–16]^

Nondrug therapies is one of the treatments recommended by various guidelines for KOA.^[17,18]^ Current trials show that massage therapy is an effective nondrug intervention,^[19]^ which is possible to be more flexible in dealing with different clinical manifestations of CP in patient with KOA. There is evidence that massage has a positive effect on the treatment of KOA symptoms, such as pain without a specified course, stiffness and dysfunction.^[20]^ Massage does not have any major risks or side effects, and has the characteristics of high security, low cost, and convenient operation,^[21]^ and it has certain advantages for patients with drug allergy.^[22]^ This systematic review will attempt to provide a basis for evaluating the credibility of massage therapy in patients with CP caused by KOA.

Meta-analysis is a powerful statistical technique and is widely accepted as an important tool of evidence-based medicine. So far, there is no evidence for massage to be used for CP in patients with KOA for more than 6 months. Therefore, we perform this protocol to comprehensively assess the effect of massage for CP in patients with KOA, which will attempt to provide a basis for evaluating the credibility.

## 2 Methods

### 2.1 Study registration

This protocol was registered on the International Platform of Registered Systematic Review and Meta-analysis Protocols (INPLASY) on December 19, 2021 (registration number: INPLASY2021120087). We will strictly perform this protocol by following the Preferred Reporting Items for Systematic Reviews and Meta-analysis Protocol statement guideline.

### 2.2 Criteria for included studies

We will conduct a comprehensive search of China National Knowledge Infrastructure (CNKI), WanFang, PubMed, EMBASE, PsycINFO, and the Cochrane Library databases from their inception to December 2021.

The inclusion criteria are as follows:

- Only randomized controlled trials (RCTs) about massage for CP by KOA will be included, with language restrictions in English or Chinese. Case report, experience report, and laboratory studies will not be included.
- All patients with CP over 6 months will be included without limitation of age, race, gender, economic level, and severity.
- The interventions of experimental group will only consist of massage therapies, mainly including general massage, acupressure, Chinese massage, relaxation, manual lymphatic drainage and so on. There will be no limitation on the methods, duration, and frequency of massage.
- The interventions of control group will involve any therapy other than massage (e.g., medication, placebo, routine care, etc).

### 2.3 Outcome

Primary outcomes: Western Ontario McMaster Osteoarthritis Index pain subscore.

Additional outcomes:

- WOMAC Stiffness subscore, Physical Function subscore and global score.
- Visual analogue scale.
- A 12-item Short-Form Health Survey (SF-12).
- Pressure pain threshold.
- Numerical rating scale of soreness intensity.
- Adverse events.
- Frequency of delayed onset of muscle soreness.

### 2.4 Search strategy

We will perform a comprehensive search in PubMed, the Cochrane Library, EMBASE and 4 Chinese databases (CNKI, Wan Fang, CBMdisc, and VIP) for articles published before December, 2021. Only RCTs that used massage as the main treatment for adults with athletic injuries will be included. The Chinese and English search strategies in PubMed database are shown in Table 1. The search terms in the Chinese databases have the same meaning as those used in the English databases. There will be no language restrictions in this review.

**Table 1:** Search terms used in Pubmed database.

Search strategy
#1 Intervention: (((((((((((((((((((((((((((Massage[MeSH Major Topic]) OR (Zone Therapy)) OR (Therapies, Zone)) OR (Zone Therapies)) OR (Therapy, Zone)) OR (Massage Therapy)) OR (Massage Therapies)) OR (Therapies, Massage)) OR (Therapy, Massage)) OR (Manipulations, Musculoskeletal)) OR (Manipulation Therapy)) OR (Manipulative Therapies)) OR (Manipulative Therapy)) OR (Therapies, Manipulative)) OR (Therapy, Manipulative)) OR (Therapy, Manipulation)) OR (Manipulation Therapies)) OR (Therapies, Manipulation)) OR (Reflexology)) OR (Bodywork)) OR (Bodyworks)) OR (Rolfing)) OR (Craniosacral Massage)) OR (Massage, Craniosacral)) OR (Manual Therapies)) OR (Manual Therapy)) OR (Therapies, Manual)) OR (Therapy, Manual)
#2 Participant: ((((((((((Chronic Pain[MeSH Major Topic]) OR (Chronic Pains)) OR (Pains, Chronic)) OR (Pain, Chronic)) OR (Widespread Chronic Pain)) OR (Chronic Pain, Widespread)) OR (Chronic Pains, Widespread)) OR (Pain, Widespread Chronic)) OR (Pains, Widespread Chronic)) OR (Widespread Chronic Pains)) AND (((((Osteoarthritis, Knee[MeSH Major Topic]) OR (Knee Osteoarthritides)) OR (Knee Osteoarthritis)) OR (Osteoarthritis of Knee)) OR (Osteoarthritis of the Knee))
#3 Study design: (randomized controlled trial [pt] OR controlled clinical trial [pt] OR randomized [tiab] OR placebo [tiab] OR clinical trials as topic [mesh: noexp] OR randomly [tiab] OR trial [tiab]) NOT (animals [mh] NOT humans [mh])
#4 #1 AND #2 AND #3

### 2.5 Identification of studies

**Figure 1:**
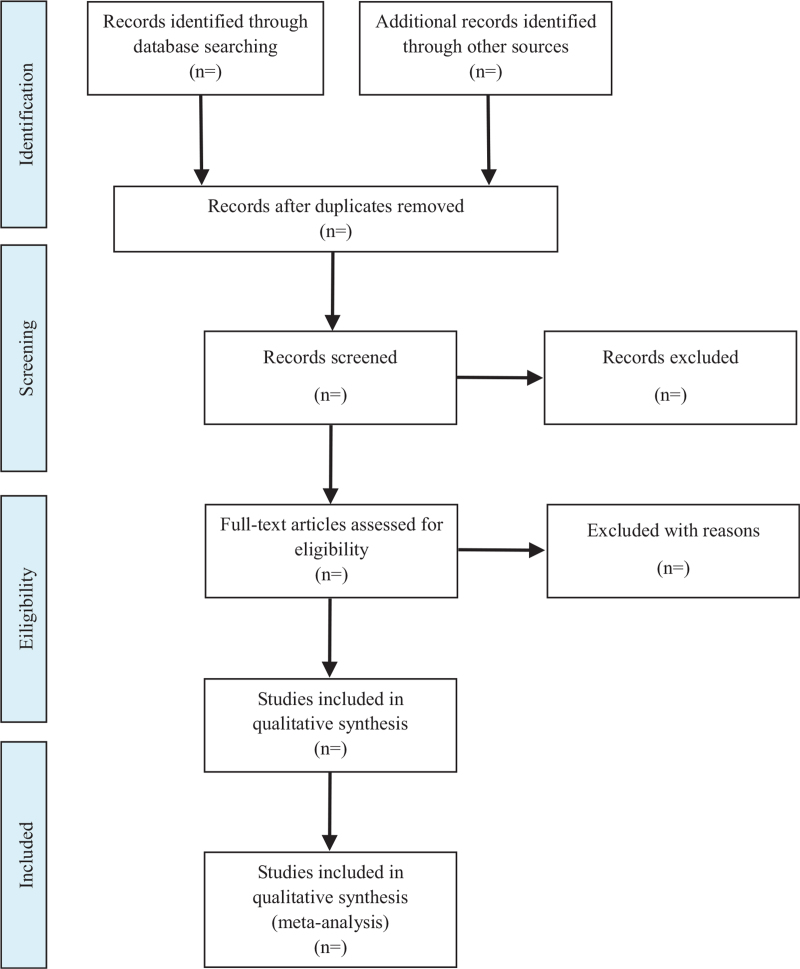
This figure is a flow diagram of study selection. A. Searching the literature in databases. B. Screening of the title and abstract of the articles. C. Choosing the eligible studies. D. Including the studies which is in qualitative synthesis.

All the search results will be imported into NoteExpress v3.5.0.9054 for management. Two reviewers (JX and YF) will independently screen all potentially eligible studies. Titles and abstracts will be screened first to exclude irrelevant citations. Full text of all the articles with potentially relevant abstracts will be retrieved and screened according to the study eligibility criteria. Disagreements will be resolved by consensus or discussion with a third reviewer (GG). The research flow chart is shown in Figure 1.

### 2.6 Data collection

Two reviewers will extract data from the included literature through Microsoft Excel 2010 (Microsoft company, Seattle, WA), mainly including the following information (Table 2):

- General information about the study, such as authors, year of publication, country, groups, sample size, age, and gender.
- Detailed treatment information, such as diagnostic criteria and parameters of intervention.
- Pain scores. Other outcome measurements, such as SF-12 or Frequency of delayed onset of muscle soreness, will be extracted if they are mentioned in the study.
- Duration of the pain.

**Table 2:** Data extraction form.

First authors	Year	Country	Sample size	Mean age
Gender	Pain location	Duration	Follow-up	Diagnostic criteria
Experimental group intervention	Control group intervention	Main outcome assessments		

### 2.7 Quality of evidence assessment

Based on Grading of Recommendations Assessment Development and Evaluation, we will assess the quality of evidence as 4 grades: high quality, moderate quality, low quality, and very low quality.^[23]^ In addition, we will use the online guideline development tool to conduct this process.

### 2.8 Risk of bias assessment

Study quality will be assessed in RevMan V5.4.1 (the Nordic Cochrane Centre, Cochrane Collaboration) using the Cochrane risk of bias tool.^[24]^ The risk of bias for each of the following domains will be assessed for each study: random sequence generation, allocation concealment, blinding of participants and personnel, blinding of outcome assessments, incomplete outcome data, selective reporting, and other bias. Each study included will be rated as having a high, low, or unclear risk of bias. Two reviewers (JX and BW) will evaluate the consistency of all the extracted data and quality ratings. Disagreements will be resolved by discussion with a third reviewer (GG).

### 2.9 Statistical analysis

Revman 5.4 software will be used to perform statistical analysis. For discontinuous variables, the risk ratio with 95% confidence interval (CI) will be selected. For continuous variables, the weighted mean difference with 95% CI will be selected when the measuring instruments are the same, and the standardized mean difference with 95% CI will be selected when the measuring instruments are different. We will use the fixed-effect model if there is no significant heterogeneity (*P* > .1 or *I*^2^ < 50%). If there is a significant heterogeneity (*P* > .1 or *I*^2^ < 50%), we will conduct subgroup analysis or sensitivity analysis to identify possible causes of heterogeneity among populations.

### 2.10 Subgroup analysis

If the necessary data are available, subgroup analysis will be conducted according to the following criteria^[25]^:

- The treatment period.
- Different acupuncture points with massage.
- Different types of manipulation (e.g., kneading, rolling, pressing).

### 2.11 Subgroup analysis

To identify the robustness of the meta-analysis, low-quality trials, with high risks of bias or outcomes that are seriously distant from the rest of the data, will be excluded.

### 2.12 Ethics and dissemination

Ethical approval will not be in need because the data used in this systematic review will not be individual patient data, and there will be no concerns regarding privacy.

## 3 Discussion

Quality of life of a large number of patients with KOA is reduced by CP.^[5]^ The mechanism of CP in KOA is complex and the degree of pain may be inconsistent with the local abnormal pathological manifestations of the knee.^[26]^ At molecular level, an animal model study shows that inflammation contributes to the evolution of joint tissue degradation and remodeling as well as joint pain.^[27,28]^ On the structural image, CP can cause more bad experiences and induce changes in brain structure and brain function related to emotion. The reorganization of functional brain network structures such as thalamus, periaqueductal gray (PAG), anterior cingulate cortex (ACC) and prefrontal cortex (PFC) is closely related to KOA-related CP.^[29–34]^ Emotion can in turn aggravate the pain experience.^[35]^ At biomechanical aspect, a study compared the walking gait biomechanics of patients with KOA after intensive intervention of lower extremities. Fifty-three patients with KOA had slight changes in their knee flexion angle (KFA) due to the enhancement of lower limb muscle strength 10 times in 28 days.^[36]^

Massage in the treatment of KOA has a history of thousands of years in China.^[37]^ The efficacy of massage in the treatment of KOA has been confirmed by some RCT.^[20,38]^ A study found that massage can change the expression of RANTES and MCP-1 and improve the symptoms of KOA,^[27]^ The latest research also points out that massage can not only remove neutrophils from seriously injured muscle tissue, but also eliminate inflammatory factors released during the process, thus promoting the regeneration of muscle fibers.^[39]^ It can also adjust the changes of pain-related brain function network.^[30,34,40]^ A study of 72 KOA patients shows that 36 times massage in 12 weeks can improve the soft tissue tension, adjust the structural relationship of the knee joint, make the tibial angle tend to normal range to balance local mechanics, reduce the degree of varus deformity and reduce the pain of patients.^[41]^ A study of 18 female patients with KOA shows that 6 weeks of massage plus exercise is more beneficial to blood circulation, muscle tension, and flexibility than only exercise,^[28]^ thus improving patients’ mood and quality of life. An increasing number of studies have demonstrated that massage has beneficial effects on symptom management in patients with KOA^[28,42]^; however, almost all the studies focused on pain without time limit. And there is a widespread lack of identification of the most effective massage treatment for CP.

Therefore, this online meta-analysis will provide a detailed summary and analysis of the latest evidence, with a focus on the available massage methods. We hope that our findings will help patients, clinicians and health care policy makers make better treatment choices for CP.

## Author contributions

JX, YF, and BW conceived the study. SX, HZ, GW, and GG provided general guidance to the drafting of the protocol. JX and BW drafted the protocol. GW and SX designed the search strategy. GG, BW, and JX drafted the manuscript. SX, HZ, BW, GW, YF, JX, and GG reviewed and revised the manuscript. All authors have read and approved the final version of the manuscript.

**Conceptualization:** Guangxin Guo.

**Funding acquisition:** Guangxin Guo.

**Writing – original draft:** Jianghan Xu, Boyi Wu, Shengji Xie, Guanghui Wu, Heng Zhang, Yangyang Fu, Guangxin Guo.

**Writing – review & editing:** Jianghan Xu, Boyi Wu, Shengji Xie, Guanghui Wu, Heng Zhang, Yangyang Fu, Guangxin Guo.

## References

[1] Bannuru RR Osani MC Vaysbrot EE. OARSI guidelines for the non-surgical management of knee, hip, and polyarticular osteoarthritis. Osteoarthritis Cartilage 2019;27:1578–89. 31278997 10.1016/j.joca.2019.06.011

[2] Allen KD Golightly YM. State of the evidence. Curr Opin Rheumatol 2015;27:276–83. 25775186 10.1097/BOR.0000000000000161 PMC4405030

[3] Murray CJ Vos T Lozano R. Disability-adjusted life years (DALYs) for 291 diseases and injuries in 21 regions, 1990-2010: a systematic analysis for the Global Burden of Disease Study 2010. Lancet 2012;380:2197–223. 23245608 10.1016/S0140-6736(12)61689-4

[4] Harper SA Roberts LM Layne AS. Blood-flow restriction resistance exercise for older adults with knee osteoarthritis: a pilot randomized clinical trial. J Clin Med 2019;8:265. 10.3390/jcm8020265 PMC6406824 30795545

[5] Hunter DJ Schofield D Callander E. The individual and socioeconomic impact of osteoarthritis. Nat Rev Rheumatol 2014;10:437–41. 24662640 10.1038/nrrheum.2014.44

[6] van Tunen J Dell’Isola A Juhl C. Association of malalignment, muscular dysfunction, proprioception, laxity and abnormal joint loading with tibiofemoral knee osteoarthritis - a systematic review and meta-analysis. BMC Musculoskelet Disord 2018;19:273. 30055600 10.1186/s12891-018-2202-8 PMC6064629

[7] Ji C Fu Q Yang Z. Research progress of community epidemiological investigation of chronic pain. Chin J Pain Med 2018;24:542–7.

[8] Lv ZT Shen LL Zhu B. Effects of intensity of electroacupuncture on chronic pain in patients with knee osteoarthritis: a randomized controlled trial. Arthritis Res Ther 2019;21:120. 31088511 10.1186/s13075-019-1899-6 PMC6518678

[9] Cross M Smith E Hoy D. The global burden of hip and knee osteoarthritis: estimates from the global burden of disease 2010 study. Ann Rheum Dis 2014;73:1323–30. 24553908 10.1136/annrheumdis-2013-204763

[10] Debono DJ Hoeksema LJ Hobbs RD. Caring for patients with chronic pain: pearls and pitfalls. J Am Osteopath Assoc 2013;113:620–7. 23918913 10.7556/jaoa.2013.023

[11] Berneath LD D’Alonzo GJ. A new look for the Journal of the American Osteopathic Association. J Am Osteopath Assoc 2013;113:06. 23329798

[12] Bonica JJ. Management of pain with regional analgesia. Postgrad Med J 1984;60:897–904. 6514649 10.1136/pgmj.60.710.897 PMC2418093

[13] Riddle DL Kong X Fitzgerald GK. Psychological health impact on 2-year changes in pain and function in persons with knee pain: data from the Osteoarthritis Initiative. Osteoarthritis Cartilage 2011;19:1095–101. 21723400 10.1016/j.joca.2011.06.003 PMC3159740

[14] Hawker GA. Experiencing painful osteoarthritis: what have we learned from listening? Curr Opin Rheumatol 2009;21:507–12. 19633560 10.1097/BOR.0b013e32832e99d7

[15] Gregori D Giacovelli G Minto C. Association of pharmacological treatments with long-term pain control in patients with knee osteoarthritis: a systematic review and meta-analysis. JAMA 2018;320:2564–79. 30575881 10.1001/jama.2018.19319 PMC6583519

[16] Torrance N Elliott AM Lee AJ Smith BH. Severe chronic pain is associated with increased 10 year mortality. A cohort record linkage study. Eur J Pain 2010;14:380–6. 19726210 10.1016/j.ejpain.2009.07.006

[17] Arden NK Perry TA Bannuru RR. Non-surgical management of knee osteoarthritis: comparison of ESCEO and OARSI 2019 guidelines. Nat Rev Rheumatol 2021;17:59–66. 33116279 10.1038/s41584-020-00523-9

[18] Osteoarthritis: care and management, London, National Institute for Health and Care Excellence (NICE); 2020. 33705083

[19] Deveza LA Nelson AE Loeser RF. Phenotypes of osteoarthritis: current state and future implications. Clin Exp Rheumatol 2019;37: (Suppl 120): 64–72. 31621574 PMC6936212

[20] Pehlivan S Karadakovan A. Effects of aromatherapy massage on pain, functional state, and quality of life in an elderly individual with knee osteoarthritis. Jpn J Nurs Sci 2019;16:450–8. 31144450 10.1111/jjns.12254

[21] Nasiri A Mahmodi MA. Aromatherapy massage with lavender essential oil and the prevention of disability in ADL in patients with osteoarthritis of the knee: a randomized controlled clinical trial. Complement Ther Clin Pract 2018;30:116–21. 29389470 10.1016/j.ctcp.2017.12.012

[22] Tang X, A systematic review of massage treatment for knee osteoarthritis, vol. Beijing University of Chinese Medicine; 2018: 55.

[23] Puhan MA Schunemann HJ Murad MH. A GRADE Working Group approach for rating the quality of treatment effect estimates from network meta-analysis. BMJ 2014;349:g5630. 25252733 10.1136/bmj.g5630

[24] Higgins J, Green SR, Chapter 8: Assessing risk of bias in included studies. Cochrane Handbook for Systematic Reviews of Interventions Version 5.1.0. Te Cochrane Collaboration, London, UK, 2011, http://www.cochrane-handbook.org.

[25] Lipkovich I Dmitrienko A Muysers C Ratitch B. Multiplicity issues in exploratory subgroup analysis. J Biopharm Stat 2018;28:63–81. 29173045 10.1080/10543406.2017.1397009

[26] Guo J Chen Y Li Z. The cerebral mechanism of acupuncture for treating knee osteoarthritis: study protocol for a randomized controlled trial. Trials 2019;20:126. 30760314 10.1186/s13063-019-3233-7 PMC6375127

[27] Wang M Liu L Zhang CS. Mechanism of Traditional Chinese Medicine in treating knee osteoarthritis. J Pain Res 2020;13:1421–9. 32606908 10.2147/JPR.S247827 PMC7304682

[28] Cortes GV Gallego IT Lazaro NI Pecos MD. Effectiveness of massage therapy as co-adjuvant treatment to exercise in osteoarthritis of the knee: a randomized control trial. J Back Musculoskelet Rehabil 2014;27:521–9. 24867903 10.3233/BMR-140476

[29] Reddan MC Wager TD. Modeling pain using fMRI: from regions to biomarkers. Neurosci Bull 2018;34:208–15. 28646349 10.1007/s12264-017-0150-1 PMC5799128

[30] Kang D McAuley JH Kassem MS Gatt JM Gustin SM. What does the grey matter decrease in the medial prefrontal cortex reflect in people with chronic pain? Eur J Pain 2019;23:203–19. 30101509 10.1002/ejp.1304

[31] Coulombe MA Lawrence KS Moulin DE. Lower functional connectivity of the periaqueductal gray is related to negative affect and clinical manifestations of fibromyalgia. Front Neuroanat 2017;11:47. 28642688 10.3389/fnana.2017.00047 PMC5462926

[32] Segerdahl AR Themistocleous AC Fido D Bennett DL Tracey I. A brain-based pain facilitation mechanism contributes to painful diabetic polyneuropathy. Brain 2018;141:357–64. 29346515 10.1093/brain/awx337 PMC5837628

[33] Peng WW Tang ZY Zhang FR. Neurobiological mechanisms of TENS-induced analgesia. Neuroimage 2019;195:396–408. 30946953 10.1016/j.neuroimage.2019.03.077 PMC6547049

[34] Liao X Mao C Wang Y. Brain gray matter alterations in Chinese patients with chronic knee osteoarthritis pain based on voxel-based morphometry. Medicine (Baltimore) 2018;97:e0145. 29561420 10.1097/MD.0000000000010145 PMC5895331

[35] Boersma K Sodermark M Hesser H. Efficacy of a transdiagnostic emotion-focused exposure treatment for chronic pain patients with comorbid anxiety and depression: a randomized controlled trial. Pain 2019;160:1708–18. 31335641 10.1097/j.pain.0000000000001575 PMC6687409

[36] Davis HC Luc-Harkey BA Seeley MK Troy BJ Pietrosimone B. Sagittal plane walking biomechanics in individuals with knee osteoarthritis after quadriceps strengthening. Osteoarthritis Cartilage 2019;27:771–80. 30660722 10.1016/j.joca.2018.12.026 PMC6475608

[37] Zhang Z, A self-control study based on MRI to evaluate the influence of massage manipulation on cartilage metabolism in patients with knee osteoarthritis, vol. China Academy of Chinese Medical Sciences; 2020: 66.

[38] Perlman A Fogerite SG Glass O. Efficacy and safety of massage for osteoarthritis of the knee: a randomized clinical trial. J Gen Intern Med 2019;34:379–86. 30543021 10.1007/s11606-018-4763-5 PMC6420526

[39] Seo BR Payne CJ McNamara SL. Skeletal muscle regeneration with robotic actuation-mediated clearance of neutrophils. Sci Transl Med 2021;13:eabe8868. 34613813 10.1126/scitranslmed.abe8868 PMC8961724

[40] Qing L, Study on fMRI and rectus femoris contrast-enhanced ultrasound in the treatment of chronic knee osteoarthritis pain, vol. Beijing University of Chinese Medicine; 2019: 73.

[41] Liu K Cao X Sun K. Application of structural manipulation in the treatment of inverted knee osteoarthritis. J Liaoning Univ Tradit Chin Med 2021;23:167–70.

[42] Zhao Y Wu L Chen YF. Stiletto needle combined with massotherapy for pain in patients with knee osteoarthritis. Zhongguo Zhen Jiu 2020;40:247–50. 32270635 10.13703/j.0255-2930.20190225-0005

